# Influence of Pre-Stress Magnitude on Fatigue Crack Growth Behavior of Al-Alloy

**DOI:** 10.3390/ma11081267

**Published:** 2018-07-24

**Authors:** Chunguo Zhang, Weizhen Song, Qitao Wang, Wen Liu

**Affiliations:** 1 Key Laboratory of Road Construction Technology and Equipment, MOE, Chang’an University, Xi’an 710064, China; songwz1234@163.com (W.S.); chdwqt@163.com (Q.W.); 2 Department of Civil Engineering, Beijing Forestry University, Beijing 100083, China; liuwen@bjfu.edu.cn

**Keywords:** pre-stress magnitude, fatigue crack growth, Al-alloy, hardness

## Abstract

From tensile overload to shot peening, there have been many attempts to extend the fatigue properties of metals. A key challenge with the cold work processes is that it is hard to avoid generation of harmful effects (e.g., the increase of surface roughness caused by shot peening). Pre-stress has a positive effect on improving the fatigue property of metals, and it is expected to strength Al-alloy without introducing adverse factors. Four pre-stresses ranged from 120 to 183 MPa were incorporated in four cracked extended-compact tension specimens by application of different load based on the measured stress–strain curve. Fatigue crack growth behavior and fractured characteristic of the pre-stressed specimens were investigated systematically and were compared with those of an as-received specimen. The results show that the pre-stress ranged from 120 to 183 MPa significantly improved the fatigue resistance of Al-alloy by comparison with that of the as-received specimen. With increasing pre-stress, the fatigue life first increases, then decrease, and the specimen with pre-stress of 158 MPa has the longest fatigue life. For the manner of pre-stress, no adverse factor was observed for increasing fatigue property, and the induced pre-stress reduced gradually till to disappear during subsequent fatigue cycling.

## 1. Introduction

Al-alloys have increasing applications in the fields of vehicles, high-speed rail and aerospace due to their high strength-to-weight ratio, outstanding ductility, and good formability [1,2]. To further improve their mechanical properties, especially fatigue performance, a number of cold working processes, e.g., shot peening and tensile overload, have been attempted to improve surface microstructure and/or to induce compressive residual stresses in metals.

Shot peening, the most commonly used method, introduces compressive residual stresses in the surface layer because plastic deformation is constrained by adjacent deeper material, which can modify the mechanical properties of metals [3,4,5,6]. Yet, shot peening influence is closely pertinent to the applied process parameters such as peening intensity, particle size and material, and nozzle distance [7,8]. As is well known, a deeper layer with higher compressive residual stresses has a more effective effect on improving fatigue properties. For that reason, a number of cutting-edge techniques, e.g., laser shock peening [9,10], ultrasonic peening [11], ultra-fine particle peening [12], and deep rolling treatment [13] have been developed and adopted to get a deeper layer with higher compressive residual stresses [14,15].

The main target of shot peening is to increase the fatigue crack initiation life of metals such as Al alloys. However, the surface roughness after shot peening increases with the increase in the density of shot peening and the depth of the residual-stress layer [16], which accelerates the nucleation and propagation of fatigue crack instead. Besides that, residual stress relaxation in a shot-peened specimen during fatigue cycling is inevitable, and the problem is getting worse with the increase of the stress amplitude [17]. Even for giga-cycle regimes (10^9^ cycles), the fatigue strength of Al-alloy is increased by only 20–30% due to the application of shot peening [18]. Another problem is that the shot peening-induced residual stresses are significantly influenced by initial residual stresses distribution of materials [19], which makes their applications more complex and more uncertain. That is, shot peening technologies generate both beneficial and harmful influences on improving fatigue properties, and it is hard to balance them. For example, softer metal has a greater work-hardening capacity in comparison with harder metal [20], but has a lower capacity for generating residual stresses.

Tensile overload, another commonly used cold work process, has similar fundamental questions as the shot peening. As well known, the change of fatigue crack growth rate da/dN after application of a single tensile overload usually contains three stages: initial acceleration, subsequent retardation, and final recovery [21,22,23]. The da/dN at the stages of retardation and recovery is lower than the corresponding normal value, which is beneficial for increasing fatigue life of metals. Due to the occurrence of initial da/dN acceleration, however, the fatigue life extension is limited significantly [24,25].

The main aim of this paper was to address the improvement of the fatigue performance of 5052 Al-alloy, which has been widely used in vehicle and aerospace industries. The influence rule of pre-stress magnitude on the fatigue crack growth, life extension, and fatigue-fractured characteristic was investigated systematically. A pre-stress was incorporated by application of a statistic load after introducing a fatigue pre-crack of approximate 1 mm. The different pre-stresses were incorporated by changing the magnitude of the applied static load.

## 2. Material and Experiments

The material used in this study was 5052 Al alloy, which is widely used in engineering structures subjected to fatigue loading. A standard extended-compact tension (E-CT) specimen with a U-notch and with a thickness of 10 mm was adopted for fatigue test, and the rolling direction of the plate was perpendicular to the direction of the Mode-I crack propagation path. All specimens were machined from the same plate using wire-electrode cutting.

**Figure 1:**
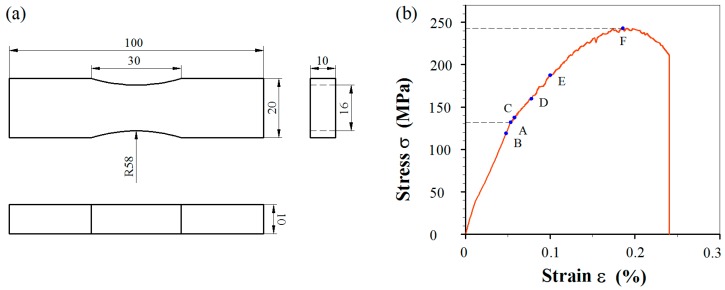
(**a**) The geometry and detailed dimensions of the Al-alloy specimen for measuring the stress–strain curve (in mm); and (**b**) the experimental result.

To determine the magnitude of pre-stress, the stress–stain curve of 5052 Al-alloy was measured by using a servo-hydraulic universal machine (TENSON-WDW-T100, Jinan Tianchen Testing machine Manufacture Co. Ltd., Jinan, China) with a crosshead speed of 5 mm/min until fracture on a specimen (Figure 1a). The measured mechanical properties are listed in Table 1.

The marked points in Figure 1b correspond to σ_y_ (A), approximate (1 ± 0.1)σ_y_ (B & C), 1.2σ_y_ (D), 1.4σ_y_ or 0.76σ_UTS_ (E) and σ_UTS_ (F), respectively, which are summarized in Table 2 together with applied loads for the incorporation of pre-stresses. There are five fatigue specimens in total, of which four are pre-stressed and one is as-received.

**Figure 2:**
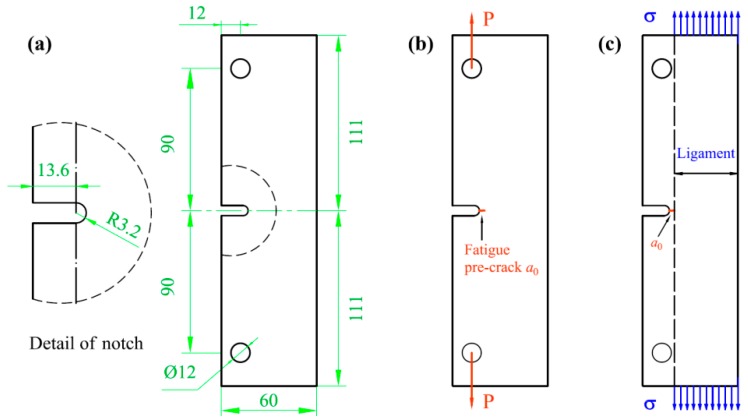
The fabrication of a pre-stressed specimen for fatigue testing: (**a**) E-CT specimen (in mm); (**b**) specimen with a fatigue pre-crack; and (**c**) application of pre-stress.

Before fatigue testing, as illustrated in Figure 2, a fatigued pre-crack of approximate 1 mm from the U-notch root was firstly introduced with gradual increasing Δ*K* in each specimen so that the initial crack length after fatigue pre-cracking is approximate 5.8 from the loading line. Then a pre-stress was introduced carefully over the un-cracked ligament of each specimen by using a servo-hydraulic universal machine and holding for 30 min after reaching the peak load. Finally, all the four pre-stressed specimens and the as-received specimen were loaded under the same fatigue loading conditions: constant amplitude loading (maximum load ≈ 12 kN) with haversine waveform at a frequency of 2 Hz was used, and the R ratio was set at near zero (approximate 0.04) during all the fatigue crack propagation tests.

Fatigue crack growth tests were performed on cracked E-CT specimens with and without pre-stresses using a servo-hydraulic fatigue test machine with a capacity of 100 kN. Paris curves, i.e., da/dN-Δ*K* curves, are shown in the following sections, where the intensity stress intensity Δ*K* is calculated with the following formula [26]:

(1) $$K=\frac{\left[P/(B\sqrt{W})]\times (2+\alpha \right)}{\left[{\left(1-\alpha \right)}^{3/2}\times {\left(1-d/W\right)}^{1/2}\right]}\times (1.15+0.94\alpha -2.48\alpha^{2}+2.95\alpha^{3}-1.24\alpha^{4})$$

where *P* = the applied maximum load, *B* = the thickness, *W* = the width of E-CT specimen, *a* = the crack length from the loading line, *d* = the distance from edge to loading line, *α* = *a*/(*W* − *d*), and 0.1 ≤ (*a* + *d*)/*W* < 1.

After fatigue testing, half of each fractured sample were examined carefully with a scanning electron microscopy (SEM, TESCAN, Brno, Czech Republic) to characterize the fracture mechanism.

## 3. Results

### 3.1. Effect of Pre-Stress Magnitude on Fatigue Property

**Figure 3:**
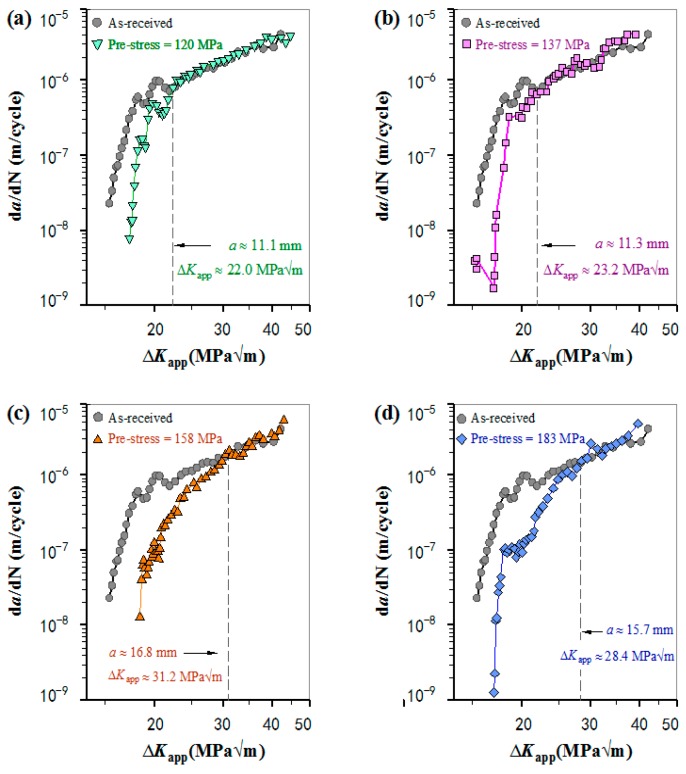
The da/dN-Δ*K*_app_ curves for the comparison between the diverse pre-stress specimens and the as-received specimen: (**a**) the as-received specimen and the specimen with pre-stress = 120 MPa; (**b**) the as-received specimen and the specimen with pre-stress = 137 MPa; (**c**) the as-received specimen and the specimen with pre-stress = 158 MPa; and (**d**) the as-received specimen and the specimen with pre-stress = 183 MPa.

In Figure 3, Paris curves are plotted in terms of da/dN as a function of the applied stress intensity factor Δ*K*_app_ for the pre-stressed specimens, and compared with that of the as-received specimen. It is evident that, after introducing pre-stress, the da/dN decelerated significantly by showing much lower da/dN in comparison to that of the as-received specimen and then increased gradually to a corresponding normal value. This indicates that pre-stress has a significant influence on improving the fatigue resistance of Al-alloy at the beginning, and the positive influence fell gradually with the increase of fatigue cycling number and/or crack length. This is because the compressive residual stresses are partially relaxed during fatigue test [17], which gradually reduces the degree of improvement on fatigue crack growth behavior.

Further analysis found that the degree of da/dN deceleration after application of pre-stress is closely pertinent to the magnitude of pre-stress. The vertically dotted line in each figure marks the point at which the da/dN recovered to a normal level, and gives the corresponding crack length *a* and Δ*K*_app_. The length of the pre-stress affected zone, i.e., the distance from the point of pre-stress application to the point at which da/dN recovers to a normal level, increases with increasing pre-stress magnitude. The fatigue cycles elapsed during the crack propagated through the pre-stress affected zone increases first then decrease with the increase of pre-stress magnitude, and the peak value occurs at pre-stress ≈ 158 MPa. These results for all the four pre-stressed cases are summarized in Table 3.

**Figure 4:**
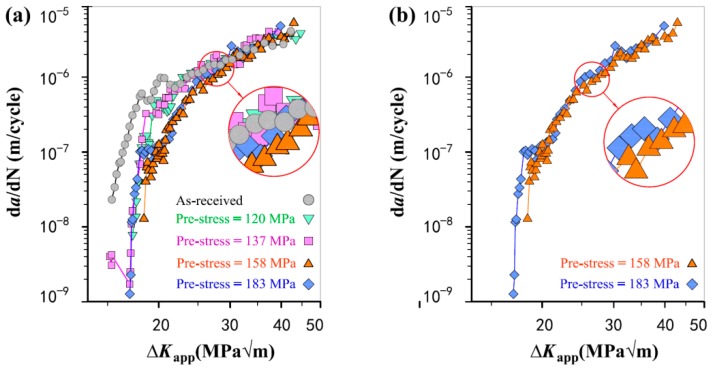
The da/dN-Δ*K*_app_ curves for direct comparison (**a**) between the four pre-stressed specimens and the as-received specimen; and (**b**) between 158 MPa pre-stress and 183 MPa pre-stress.

To make a direct and intuitionistic comparison to emphasize the influence of pre-stress magnitude, the fatigue crack growth curves of the four pre-stressed specimens and the as-received specimen are plotted together as illustrated in Figure 4. It is evident that the higher pre-stress results in slower fatigue crack growth and thus increases fatigue resistance when the pre-stress varies from 120 to 158 MPa. However, the specimen with a higher pre-stress of 183 MPa exhibits a reduction of improvement in fatigue resistance by showing a higher da/dN in comparison to that of the specimen with a pre-stress of 158 MPa. As a result, the overall improvement in fatigue life is not as significant as in the case of 158 MPa. The direct comparisons of the different pre-stress values further suggest the pre-stress cannot be too high, and the incorporation of pre-stress around 158 MPa is optimal to improve the fatigue properties of 5052 Al-alloy.

### 3.2. Observations of Fatigue-Fractured Surfaces

**Figure 5:**
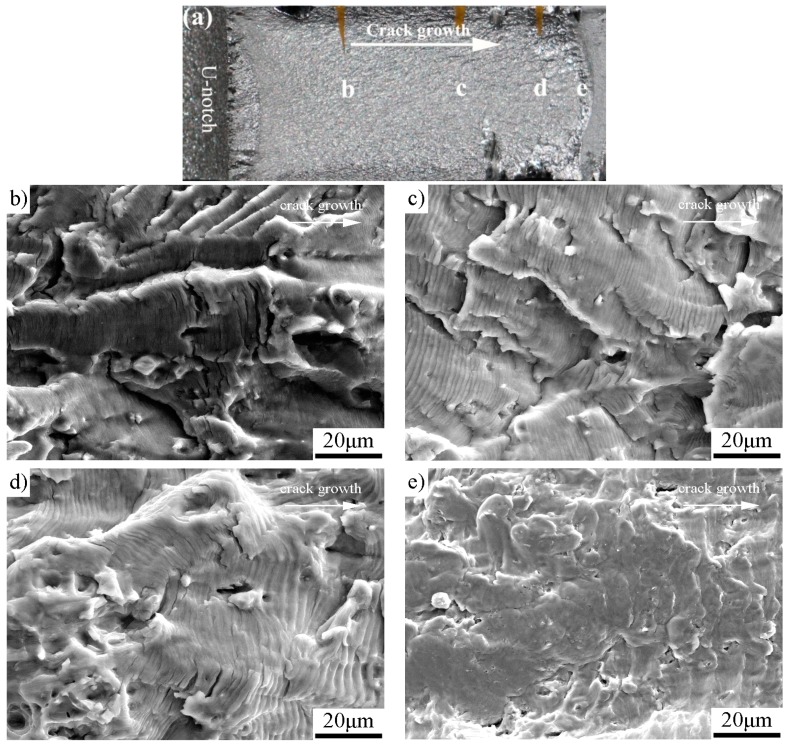
The as-received specimen: (**a**) macrograph showing the detailed SEM locations; (**b**) Δ*K*_app_ ≈ 20 MPa$\sqrt{m}$; (**c**) Δ*K*_app_ ≈ 30 MPa$\sqrt{m}$ (striation spacing ≈ 1.7 μm); (**d**) Δ*K*_app_ ≈ 40 MPa$\sqrt{m}$ (striation spacing ≈ 3.2 μm); and (**e**) unstable propagation zone.

**Figure 6:**
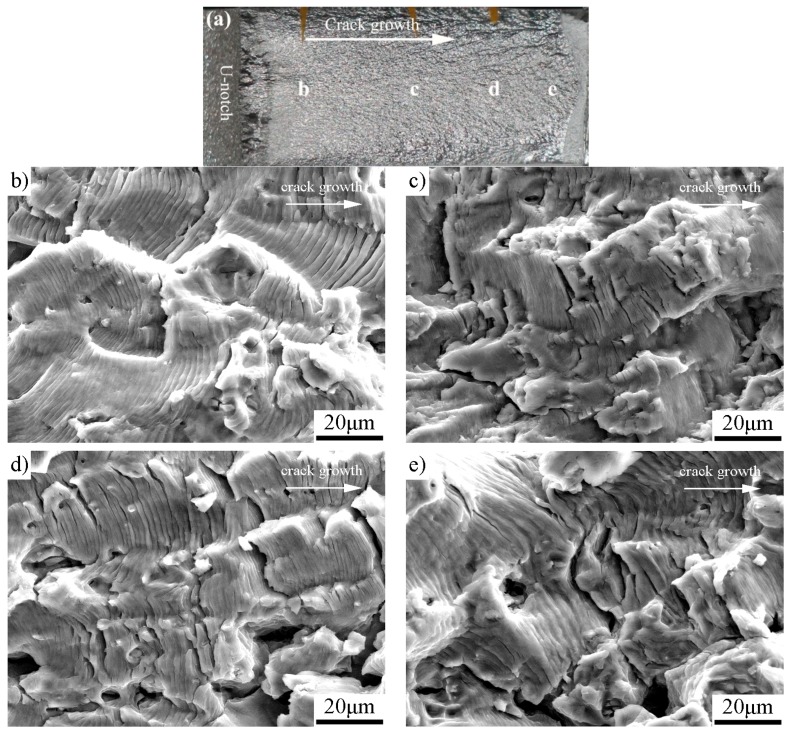
The pre-stressed specimen with pre-stress of 137 MPa: (**a**) macrograph showing the detailed SEM locations; (**b**) Δ*K*_app_ ≈ 20 MPa$\sqrt{m}$ (striation spacing ≈ 0.3 μm); (**c**) Δ*K*_app_ ≈ 30 MPa$\sqrt{m}$ (striation spacing ≈ 1.7 μm); (**d**) Δ*K*_app_ ≈ 40 MPa$\sqrt{m}$ (striation spacing ≈ 3.8 μm); and (**e**) unstable propagation zone (striation spacing ≈ 4.7 μm).

**Figure 7:**
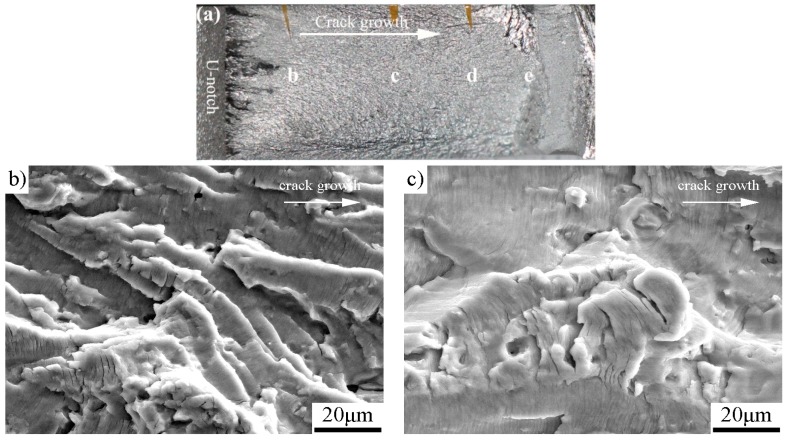

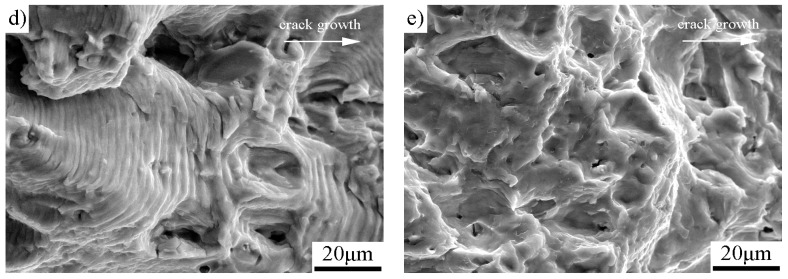
The pre-stressed specimen with pre-stress of 158 MPa: (**a**) macrograph showing the detailed SEM locations; (**b**) Δ*K*_app_ ≈ 20 MPa$\sqrt{m}$ (striation spacing ≈ 0.2 μm); (**c**) Δ*K*_app_ ≈ 30 MPa$\sqrt{m}$ (striation spacing ≈ 1.4 μm); (**d**) Δ*K*_app_ ≈ 40 MPa$\sqrt{m}$ (striation spacing ≈ 3.2 μm); and (**e**) unstable propagation zone.

**Figure 8:**
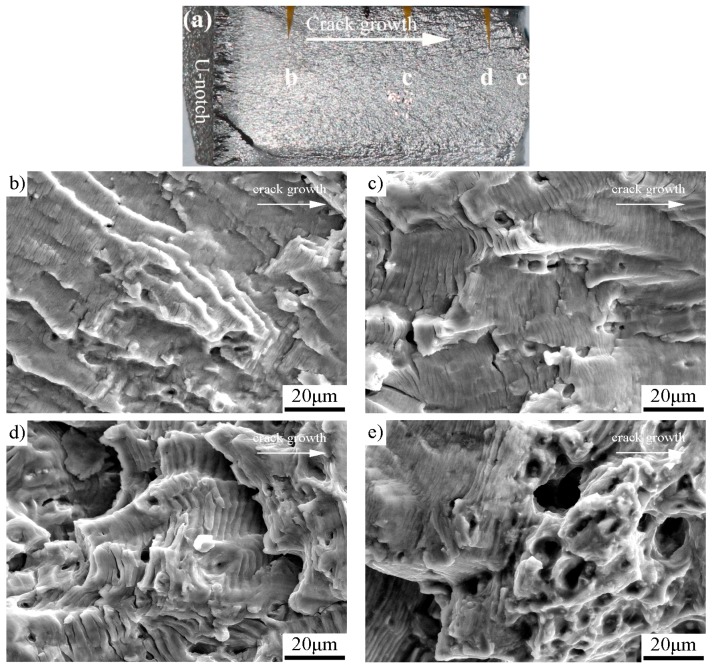
The pre-stressed specimen with pre-stress of 183 MPa: (**a**) macrograph showing the detailed SEM locations; (**b**) Δ*K*_app_ ≈ 20 MPa$\sqrt{m}$; (**c**) Δ*K*_app_ ≈ 30 MPa$\sqrt{m}$ (striation spacing ≈ 2.6 μm); (**d**) Δ*K*_app_ ≈ 40 MPa$\sqrt{m}$ (striation spacing ≈ 4.9 μm); and (**e**) unstable propagation zone.

The fatigue-fractured characteristics analyzed by SEM are shown in Figure 5 for the as-received specimen, Figure 6 for the specimen with pre-stress of 137 MPa, Figure 7 for 158 MPa, and Figure 8 for 183 MPa, respectively. To correlate the SEM observations to the relevant locations and corresponding da/dN measurements, macrographs of the fracture surfaces are numbered accordingly for each specimen. Because the pre-stressed specimens with 120 MPa and 137 MPa have similar fracture characteristics, here we only choose the case of 137 MPa to shown SEM observations. For purpose of comparison, each fractured surface shots four SEM micrographs which correspond to Δ*K*_app_ ≈ 20 MPa$\sqrt{m}$, 30 MPa$\sqrt{m}$, 40 MPa$\sqrt{m}$ and within unstable propagation zone, respectively. For all the SEM micrographs, the fatigue crack propagated from left to right.

The SEM micrographs in Figure 5, Figure 6, Figure 7 and Figure 8 show that the fatigue fracture characteristic is basic fatigue striation in both the as-received and the pre-stressed specimens, and the striation spacing becomes narrower after the incorporation of pre-stress at the beginning of fatigue crack growth. For the pre-stressed specimens, a higher pre-stress results in narrower fatigue striation spacing when the pre-stress changes between 120 and 158 MPa, but the striation spacing increases instead when the pre-stress further increases to 183 MPa in comparison to the pre-stress of 158 MPa.

## 4. Discussions

The effect and changing laws of pre-stress magnitude on fatigue crack growth of the Al-alloy were studied by the measurement of da/dN and detailed fracture characteristics. In Figure 3, the da/dN reduces significantly after the incorporation of pre-stress in all the four cases, and then gradually recovers to a normal level of da/dN (i.e., the corresponding da/dN of the as-received specimen). In other words, the fatigue performance of Al-alloy after the introduction of pre-stress subjected to cycle loading was developed by two stages: the initial improvement stage and subsequent normal stage.

It is accepted that cold working processes (e.g., shot peening and tensile overloading) the improves the fatigue resistance of metallic materials by generations of effective compressive residual stress and crack-tip blunt [27,28]. As for the methodology of pre-stress, pre-stress or compressive residual stress is the most important or predominant factor in improving the fatigue property due to crack-tip blunt does not occur. That is, compressive residual stress introduced in the un-cracked ligament of the sample reduces the effective stress intensity factor Δ*K_eff_* for crack growth, i.e., Δ*K_eff_* < Δ*K*_app_. As well known, higher pre-stress has a more effective influence to reduce the da/dN of a specimen. At the first stage or initial improvement stage, however, the degree of improvement in da/dN first increases then decreases along with the increasing of pre-stress magnitude, and the maximum occurs at a pre-stress of 158 MPa as shown in Figure 3 and Figure 4. This is because the excessive increase in pre-stress magnitude generates strain hardening. The reduction in ductility is harmful to fatigue resistance. Here, it should be pointed out that the residual stress or pre-stress distribution along the crack growth path in the un-cracked ligament is not absolute uniform because of the stress concentration at the fatigue pre-crack tip. The residual stress in front of the pre-crack tip is slightly higher than the calculated engineering stress. That is why the pre-stress of 120 MPa, which is lower than the yield strength of the Al alloy, still has an obvious influence on improving the fatigue performance of the specimen.

**Figure 9:**
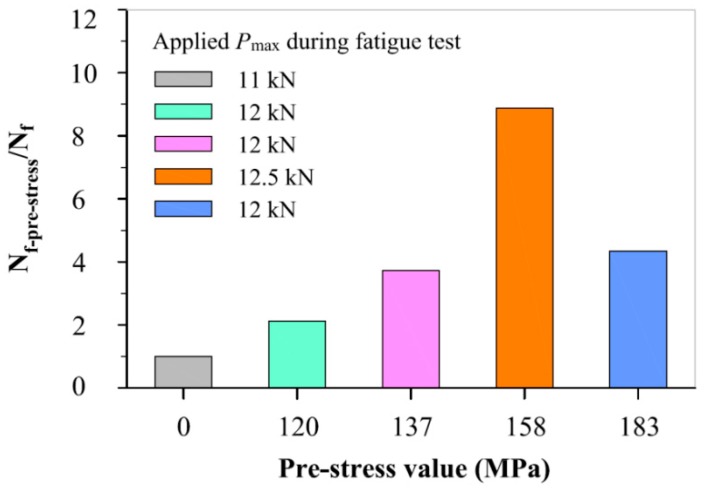
The relationship between the pre-stress magnitude and the ratio of N_f-pre-stress_ and N_f_.

To quantify the relationship between pre-stress magnitude and degree of improvement in fatigue life, Figure 9 shows the ratio of N_f-pre-stress_ (number of fatigue cyclesto failure for a specimen with pre-stress) and N_f_ (number of fatigue cycles to failure for the as-received specimen) from the same crack length. Although the specimens with pre-stresses have differently initial pre-crack (from 0.9 mm to 1.9 mm), their fatigue crack growth lives can be compared with the corresponding fatigue crack growth life of the as-received specimen because the pre-crack length in the as-received specimen is 0.9 mm. It is evident that, with an increase of the pre-stress magnitude, the degree of improvement in fatigue life increases firstly, reaches a local maximum (increases by approximate 9 times than corresponding fatigue life of the as-received specimen) at a pre-stress of 158 MPa, and then decreases. The result is consistent with the da/dN measurement discussed above. This is because the compressive residual stress is achieved at the expense of material ductility. The overall improvement in fatigue performance decreases instead if the pre-stress is excessive.

A similar relation between overload magnitude and its improvement on fatigue properties is also established in the study of a single tensile overload. To our best knowledge, it is the first time that the fatigue performance improvement is linked to the pre-stress magnitude specified by the ratio of N_f-pre-stress_ and N_f_.

In Figure 3, the Paris curves of the as-received and each pre-stressed specimen intersect at a point, which is the turning point from the improvement stage to normal stage. The turning points in the four causes differ from each other due to the difference in pre-stress magnitude. In Table 3, with increasing the pre-stress magnitude, the pre-stress affected zone increases monotonously, but the fatigue life by considering the number of fatigue cycles from comparable crack length to their respective turning point increases firstly, reaches a peak value at pre-stress of 158 MPa, and then decreases. After the turning point, the da/dN of pre-stressed specimens recovers to a normal level. It is customary that the introduced pre-stress is basic uniform stress in the un-cracked ligament of a specimen. The occurrence of the normal stage after the incorporation of pre-stress is due to the relaxation of pre-stress with increasing the number of fatigue cycling and/or crack length [14,19].

The fatigue fracture characteristics at comparable Δ*K*_app_ (20, 30 and 40 MPa) found that: (1) Pre-stress does not change the fracture mechanism of Al-alloy, which is basic fatigue striation. (2) Before the turning point (e.g., at Δ*K*_app_ ≈ 20 MPa), the fatigue striation spacing in each pre-stressed specimen is narrower than the corresponding striation spacing of the as-received specimen. (3) At the stage of initial improvement, the specimen with a pre-stress of 158 MPa has the narrowest striation spacing than those of both the as-received specimen and other pre-stressed specimens. (4) After the respective turning point, the striation spacing and unstable propagation through micro-void coalescence in the pre-stressed specimens are similar to those of the as-received specimen. The results are consistent with the da/dN measurements.

## 5. Conclusions

The influence rule of pre-stress magnitude on the fatigue crack growth, life extension, and fracture mechanism/characteristic of the 5052 Al-alloy were investigated systematically. The main conclusions were drawn as follows:

(1) The fatigue crack growth property of the Al-alloy after the introduction of pre-stress subjected to cycle loading was developed by two stages: the initial improvement stage and subsequent normal stage.
(2) Various pre-stress significantly improved the fatigue performance of Al-alloy, and no adverse factor was observed for increasing fatigue property. Pre-stress does not change the fatigue fracture characteristic (i.e., fatigue striation), but obviously reduces the fatigue striations spacing.
(3) At the stage of initial improvement, the fatigue life and crack growth resistance of a specimen increases firstly, reaches a peak value at a pre-stress of approximate 158 MPa, and then decreases, with the increasing pre-stress magnitude. This is because excessive pre-stress generates the phenomenon of strain hardening.
(4) Paris curves of the as-received and each pre-stress specimen intersect at a point, which is the turning point from the improvement stage to normal stage. The occurrence of the normal stage after incorporation of pre-stress is due to the relaxation of pre-stress with the increasing number of fatigue cycling and/or crack length.

## Figures and Tables

**Table 1:** The measured mechanical properties of 5052 Al alloy.

Material	State	Yield Strength σ_y_	Ultimate Tensile Strength σ_UTS_	Young Modulus
Al5052	H32	132 MPa	243 MPa	69.3–70.7 GPa

**Table 2:** The pre-stresses applied to Extended-compact tension (E-CT) samples for fatigue testing.

Sample	Fatigue Pre-Crack (mm)	Ligament (mm^2^) (Length × Thickness)	Applied Load (kN)	Pre-Stress (MPa) (Point in Figure 2b)
1	0.9	42.3 × 10	-	-
2	1.4	41.8 × 10	50	120 (B)
3	1	42.2 × 10	58	137 (C)
4	1.9	41.3 × 10	65.2	158 (D)
5	0.9	42.3 × 10	77.3	183 (E)

**Table 3:** The length of the pre-stress affected zone and the corresponding fatigue lives.

Pre-Stress (MPa)	120	137	158	183
Length of pre-stress affected zone (mm)	4.9	5.5	9.9	10.1
Fatigue pre-crack (mm)	1.4	1.0	1.9	0.9
Crack length recovering to normal da/dN	6.3	6.5	11.8	11.0
Number of fatigue cycles elapsed	109,055	209,857	333,650	242,905
Δ*K*_app_ ($\mathrm{MPa}\sqrt{m}$) ↔ normal da/dN	22.0	23.2	31.5	28.4
The applied maximum load *P*_max_ (kN)	12.0	12.0	12.5	12.0
